# Being in-between; exploring former cult members’ experiences of an acculturation process using the cultural formulation interview (DSM-5)

**DOI:** 10.3389/fpsyt.2023.1142189

**Published:** 2023-09-13

**Authors:** Cecilia Hadding, Olof Semb, Arja Lehti, Martin Fahlström, Mikael Sandlund, Valerie DeMarinis

**Affiliations:** ^1^Unit of Professional Development, Department of Clinical Science, Umeå University, Umeå, Sweden; ^2^Unit of Psychiatry, Department of Clinical Science, Umeå University, Umeå, Sweden; ^3^Department of Public Health and Clinical Medicine, Umeå University, Umeå, Sweden; ^4^Department of Adult Psychiatry, Innlandet Hospital Trust, Sanderud, Norway

**Keywords:** cult, coersive control, cultural formulation, acculturation, consultation

## Abstract

**Objective:**

To explore the experiences of acculturation into secular Swedish society of former members of cults, with particular focus on mental health, needs and resources.

**Design:**

Qualitative method using the Cultural Formulation Interview (CFI) from the DSM-5 as an interview guide. Analysis of participants’ experiences of acculturation through systematic text condensation.

**Participants:**

Eleven Swedish former members of ideological or religion-based cults.

**Setting:**

Swedish mainstream, secular society.

**Results:**

Former cult members experience an ‘in-between time’ in the period after leaving the cult and find themselves in a confusing, chaotic state. They describe having lived in an honor culture where acts of violence were normalized. In the cult, they felt disconnected from themselves, and post-cult they try to regain access to their own values and feelings as well as create new bonds with family members and friends outside the cult. They find it hard to talk about their cult background and find relief in communicating with other former cult members. In their post-cult life, they eventually start seeing the world in a brighter, more hopeful way than before. However, they are also at risk of re-experiencing cult-related traumatic events and of new traumatic experiences within the post-cult acculturation process, and of persistent psychological distress.

**Conclusion:**

Former cult members face a challenging acculturation process, having lost a functioning worldview upon leaving the cult but not yet gained another to take its place. While the in-between time is often transient, they may need support from the healthcare system, especially regarding mental health concerns, while establishing themselves into mainstream society.

## Introduction

All cultures, at group as well as societal level, have an influence on how a person interprets, expresses, and manages illness and related needs (1). This makes it important for healthcare providers, as members and representatives of mainstream society, to explore and understand perceptions and needs around illness as integral parts of a patient’s cultural worldview (2). Incorporating cultural awareness into healthcare consultations can facilitate access to important information that is necessary for all phases of diagnostic and treatment processes (3). During acculturation, an individual from one culture faces the challenges and opportunities of acquiring a new culture and a new worldview. Acculturation always includes interaction between the individual and society, and can have various outcomes. If an individual in an acculturation process is caught between cultural worldviews – functionally rejecting the old while not yet understanding or adjusting to the new – marginalization may occur. Experiencing marginalization makes the individual susceptible to mental health problems (4, 5).

Although the cult as a concept is widely recognized, there is no consensus regarding its definition or inclusion/exclusion criteria. In this study, a cult is defined as a group or community that uses coercive control over its members, rather than by connection to a particular religion or faith. This is a functional definition with a focus on the psychological and psychosocial aspects and effects associated with cult membership. In line with this, Langone and West (6) suggest the following definition of a cult: ‘A group or movement exhibiting a great or excessive devotion or dedication to some person, idea, or thing and employing unethically manipulative techniques of persuasion and control […] designed to advance the goals of the group’s leaders, to the actual or possible detriment of members, their families, or the community.’

A cult, in this study, can further be understood as a wholly or partially isolated context within a locality, resulting in conflictual identities where the identity of the cultic community differs from that of the surrounding cultural context or community. This presents a potential problem when the individual wants to leave the community. Previous studies on individuals leaving cults have shown that they often face difficult consequences, e.g., signs of emotional, cognitive, social and other problems (7), as well as dissociation and suicidal ideations (8).

In cults, members are typically isolated from the outside world (9, 10). Members are inducted into the strict rules of the community, paving the way for experiences of shame, created dependency, and installed phobias (11). Installed phobias include a perception of the surrounding society as not only different, but threatening or judgmental, thus making the isolation necessary. Someone who has grown up or lived for several years in a cult has been immersed in its culture, structure and worldview. The harmful component in a cult community is therefore much to do with the psychological processes of coercive control described by Hassan (9) and Lifton (12), among others.

Thus, in this study, a cult is defined as a closed cultural context where rules and coercive control of the individual have a potentially harmful effect. This definition has mainly to do with the conditions of totalitarian rule and isolation described above, rather than faith or ideology *per se*.

Sweden has no legal or otherwise official definition regarding cults. There is, however, an ongoing discussion on cults as a concept. Scholars in sociology and religious studies sometimes argue that the use of terms like ‘manipulation’ and ‘indoctrination’ when discussing cults is intended to criticize religion and specific religious movements (13). However, many healthcare professionals, as well as non-governmental organizations (NGOs) that work with former cult members, argue for a more functional view of cults as described above (14, 15).

Closed religious or ideological cultic communities that use coercive control can exist within a wider secular social context. Members of such communities experience daily life according to the community’s cultural worldview. If circumstances arise where the individual no longer belongs to that closed community and its worldview, but does not yet have a new worldview that functions in their daily life, their worldview function can be impaired. Understanding this dysfunction in worldview, and its palpable impact on the ability to address everyday challenges, can be an important part of treatment strategies for former cult members (2, 16, 17). In essence, the patient needs to find ways to acculturate into the larger social context and negotiate a new way of belonging, as well as a new way of making meaning in their life, and move towards creating a new cultural worldview for themselves.

Sweden, the setting of the present study, is highly secularized, with most Swedes scoring high on the values ‘openness’ and ‘free choice,’ according to the World Values Survey (18). It is therefore the norm to be cautious about discussing existential matters and religion. In the Nordic countries, healthcare professionals are generally not trained or prepared to discuss existential or cultural issues in a clinical setting as a means to gain a more accurate understanding of the patient’s way of interpreting their illness (2). DeMarinis (16) presents an overview of the different types of worldviews that exist in Sweden, and underlines the need for clinicians, not least in the field of mental health, to gain access to information about the patient’s worldview that is directly associated with the patient’s experience of presenting problems and symptoms.

The Diagnostic and Statistical Manual of Mental Disorders (DSM-5) (19) defines culture as: ‘…systems of knowledge, concepts, rules, and practices that are learned and transmitted across generations [...]. Cultures are open, dynamic systems that undergo continuous change over time; in the contemporary world, most individuals and groups are exposed to multiple cultures, which they use to fashion their own identities and make sense of experience.’ This definition describes culture in a way that can be applied both to particular groups and to wider societies. In this study, both perspectives are necessary. This is important, as the particular groups in focus (cults) have many cultural components that are in stark contrast to society at large, and since this definition explains the role of culture for the individual’s identity and how they make sense of experiences and form a worldview.

This study is focused on the process of transitioning from a strictly defined, closed culture to another (mainstream) culture, a process whereby the individual is required to eschew the cult’s cultural worldview that is no longer functional or valid in their new context. The attempt to create a new cultural worldview, under these circumstances, can therefore be described as an acculturation process.

## Aim

To explore the experiences of acculturation into secular Swedish society of former members of cults, with particular focus on mental health, needs and resources.

## Method

The study used a qualitative approach and was conducted *via* semi-structured interviews using the Cultural Formulation Interview (CFI) (19). The CFI is used to evaluate, highlight and examine the individual patient’s perspective on health and illness according to the patient’s cultural context and their life experiences. It considers the patient’s cultural identity and cultural explanations, and how they interpret their illness and seek to understand their problems on both a group and societal level (20). The CFI covers four main areas: (1) cultural definition of problem; (2) cultural perceptions of cause, context, and support; (3) cultural factors affecting self-coping and past help seeking; and (4) cultural factors affecting current help seeking.

In this study the CFI was used as a semi-structured interview guide for research with a vulnerable population. This use differs from clinical situations where the CFI is an aid for diagnosis and treatment planning. The questions were used to allow the respondents to describe experiences from their present and past life situations. The interviewer ensured that all areas and questions of the CFI were covered in the interview. If the respondent described something in answering one question that connected to another question in the CFI, the interviewer followed the narrative response and did not interrupt. The first author (CH), who is both a resident physician in psychiatry and a researcher, conducted all the interviews.

### Recruitment and sampling

Inclusion criteria were being aged 18 years or older and self-identifying as a former cult member. Recruitment was carried out *via* advertising on social media, i.e., Facebook, and by snowball sampling (21). The respondents replied to the research team *via* email. The research team informed all the respondents about the study *via* written information and telephone calls. No one who responded to the ads was denied participation.

Eleven respondents participated in the study, two males and nine females. Seven of them were born and raised in a cult context. Four of them had joined during their childhood/adolescence with their families or as adults. The mean time spent in their respective cults was 16 years (median 18 years). The time that had elapsed since they left ranged from 1 to 46 years (median 11 years). The cults they had left were ideology-based or religious communities. In this study, an ideology-based cult could be based on politics, therapy/personal development or yoga/meditation practices, while the religious cults were based on belief in one or several gods or god-like characters. According to the respondents, nine were based more on religion and two based more on ideology. Specific cults have not been mentioned in the study in order to ensure the privacy and safety of the respondents. The purpose of this study is not to define existing communities and congregations as cults. Moreover, mentioning a specific cult by name does not reveal much about types of exposure or practices in the cult, since the same cult may present differently in different locations and at different times (22).

All the respondents stated that potentially traumatic experiences had occurred in the cult. These differed from one respondent to the next. Events that occurred in the family during their childhood in the cult included incest, neglect, being locked up, being starved, being separated from their parents by the cult leader, being isolated, and social punishments such as not being allowed to talk to or being shunned by family members or other cult members. These experiences could either be the result of cult ideology or practices in the family that were condoned by the cult. Experiences reported both by respondents who had joined the cult as adults and by those who had been raised in the cult included social, emotional and/or physical abuse.

### Data collection and analysis

Nine of the interviews were conducted *via* video conference call. Two of the respondents requested interviews over the phone. Each interview lasted for around 1 h. The respondents also completed a form containing questions on their background such as when they had left the cult, how much time they had spent in the cult, whether they had been born and raised in the cult, and what kind of cult they had belonged to. The interviews were recorded and then transcribed verbatim. When 11 interviews had been conducted and transcribed, the data were considered rich in amount and depth and a repetition of themes was noted. Therefore, a decision was made to stop data collection.

The data collected *via* the CFI interview guide were analyzed using systematic text condensation according to Malterud (23). Systematic text condensation was developed in the tradition of Giorgi and has a phenomenological foundation (24) The reason for choosing to analyze the results as a whole and not, for example, according to the four areas of the CFI was to be able to recognize former members’ experiences over time after leaving the cult – a process that this study aims to investigate. The choice of analytical methods is further discussed in the strengths and limitations section.

Systematic text condensation includes four steps of analysis, described below. The ongoing analysis of the transcribed interviews was carried out using the shared data program ATLAS.ti (25). Thus, all the authors were easily able to access and comment on the process.

Themes were collected from the transcribed interviews (step one – overall impression). These were discussed between CH, OS and AL. Meaning-building units were then collected from the material by CH (step two – meaning units). The meaning-building units were analyzed and sorted into codes in terms of their content. The meaning-building units in each code were then sorted into 3–6 smaller subcategories that contained more defined areas from the codes. The coding of meaning-building units was repeatedly discussed between VD, AL, and CH. The aim here was not to reach consensus, but rather to gain a deeper understanding of the material. The condensation was done based upon the codes and subcategories (step three – condensation). The condensation process started by summarizing the meaning-building units in each subcategory into shorter text units written in the first person. From these, a result text was written for each subcategory (step four – synthetization). These result texts strive to follow the respondents’ words and expressions as closely as possible. Quotes were then selected to be presented with each result text. All the authors contributed to writing the paper. The study follows the Standards for Reporting Qualitative Research (SRQR) (26).

### Compliance with ethical standards

The project was funded by Umeå University and the Region of Västerbotten, funds for doctoral students. All authors certify their responsibility for this paper. All procedures performed involving human participants were in accordance with the ethical standards of the institutional and/or national research committee and with the Helsinki declaration (27) and its later amendments or comparable ethical standards. The study was approved by the Ethical Review Board in Umeå (No. 2015/189-31Ö and 2016/323–3 and 2018/128–32).

Informed consent was obtained from all individual participants included in the study.

## Results

Four codes were identified during the analysis: Culture and identity, In-between time, Needs and meaning making, and Persistent problems. Each of these codes contained subcategories. The results are presented as codes and subcategories in Table 1. The quotes within the text are from the interviews and have been chosen with the intention of giving the respondents more of a voice in the article. Where the word ‘god’ is mentioned, this is understood as referring to any form of higher power. Results are derived from an analysis of all interviews, irrespective of the frequency of a code or subcategory in the interviews.

**Table 1:** Codes and subcategories.

Codes	Subcategories
Culture and identity	Positive and valuable experiences from the cult The vulnerability of living in a closed environment A culture of honor A struggle of identity and self-view Learned not to express own values
The in-between time	Chaos, crisis, and destructivity Puzzled on adopting new ideology or not Fear and clinging to former ideology Confusion regarding world view Feeling lonely and missing friends Barriers to talk about cult experiences
Needs and meaning-making	Life changing help with trauma consequences Finding other former members to exchange experiences A new sense of belonging A new relation to faith and the cult experiences
Persistent problems	General psychiatric symptoms Body-related symptoms Trauma-related symptoms

### Culture and identity

The first code relates to former cult members’ descriptions of the cult and the culture that they lived in and left, the living conditions, their sense of identity and their experience of what was normal.

Positive narratives included how they had spent their time together with others in the cult and felt that they had learned good life values. They had learned to talk to new people and to stand up for their beliefs. They also appreciated the love and respect for others that had been expressed in the group – even if this was conditional on obeying the cult’s rules.

*‘It [the cult] has destroyed a lot for me and my siblings. But at the same time, I have learned a lot. You get trained in talking to people, to strangers and to stand up for your beliefs. Even if these were almost forced opinions from the time you were a tiny kid [….] I benefit from that now.’ ID 9.*

However, they also had experienced the normalization of abuse and potentially traumatic events in the cult (see description of sample in method section). Consequences related to these experiences were a sense of insecurity and a perception of self that was influenced by both the culture of the cult and by specific events that occurred within the cult. The environment they had experienced in the cult was highly demanding and had consumed their everyday lives: Prayers, meetings, studying and recruiting new members.

*‘You worked all the time [in the cult]. [...] Even if you were not on duty there is always this thought that there are potential people that you should be recruiting. You should constantly talk about it [the cult/the mission]and try to distinguish yourself from other people,* i.e.*, in your morality, how you behave, how you speak, a lot of stuff. It really becomes everything, so it’s not something you just, ‘now I can turn off, now it’s done’. It’s around the clock.’ ID 8.*

They lived in a closed environment and culture, even if they participated in the outside world in some ways. No transgressions that were committed in the cult, not even potentially serious crimes, were reported to the police. The cult did not want any contact with outside authorities. This often allowed incest and sexual abuse to persist for a long time in families in the cult. Former cult members described feeling lonely and having no adult to turn to when they were growing up.

*‘What I get upset about is when I realize that the cult is a contributing factor to why the abuse [incest] could go on for so many years - because I was so completely isolated.’ ID 5.*

They described the cult environment as an honor culture involving frequent moral conversations and top-down governance that included rape and forced baptism.

*‘I was baptized against my will in my teens. This was because I was repeatedly raped at home and I knew that I was not a virgin. I was not pure before God. I had committed a sin. That’s how I felt.’ ID 5.*

It was hard for them to accept themselves. They described this in terms of both ‘I am not like others outside this group’ and ‘I am not sufficiently like others in this group according to the group’s standards’. In the cult, they found they struggled to do everything right according to the group’s rules. They were told by those in authority that all their unhappiness was their own fault, that they were evil or that they did not try hard enough.

*‘I used to think that I had been immoral or committed sins, because that is how they explain it in the cult. If you feel unwell it is because you are unethical or did bad things.’ ID 6.*

They described that they had been alienated from themselves in the cult and that they were not permitted to express their own feelings. After leaving the cult, it was hard for them to recognize and express their values and feelings, having learned not to do so.

*‘I probably have not been allowed to accept my feelings at all in any form. That has been stifled.’ ID 7.*

*‘You are alienated from yourself and your own feelings and your own thoughts and from your surroundings [...] So that you lose yourself, you lose your loved ones, and you lose your place in the world.’ ID 10.*

### The in-between time

Former cult members described a state of no longer being physically in the cult but not being out of it psychologically. This was a time in between two worlds and worldviews. They felt distanced from their former sense of belonging and yet did not seem to belong anywhere else, as if they were in a state of suspension or ‘being in-between.’

It took a long time before they felt that their lives had re-started. This was a period of chaos and crisis. During this time, they describe that they made suicide attempts, committed acts of self-harm or had problems with excessive alcohol use. They described being in a process of reorganizing themselves and finding a new perspective.

*‘I would describe the years after I left the cult as being in a state of shock. I was just trying to make sure that I could function’ ID 3.*

The in-between time was a period of uncertainty, when the former cult members tried to figure out whether they wanted to adopt another religion/ideology or not. In cases where such a decision was made, this took a lot of time and consideration.

*‘I have distanced myself from it [the cult ideology] completely. But I have acquired another faith, well, should I say, another religion that feels authentic to me. But it took many years’ ID 2.*

Psychologically and mentally, they clung to their former religion or ideology. Whenever they said anything negative about the cult’s ideology, they felt a strong sense of ‘I will be killed for this’.

*‘And every time I said something slightly negative about [name of the cult], I had terrible anxiety and was afraid that I would be killed on the spot; it is a very difficult process’ ID 5.*

The fear faded with time, but it took many years. It was hard for them to figure out how to think for themselves, and it took time to learn new ways of doing things. They experienced a lot of fear during this time, much of which was connected to confusion regarding their worldview, i.e., whether all people outside the cult really were evil.

*‘There is a lot of dissonance in this, but I started to see, slowly but surely, it was not true (what they had said in the cult.) Not everyone is evil. It took a very long time; it was a very long period of isolation’ ID 3.*

This was a time of loneliness. They had no one to share their feelings with, and there was a sense that nobody would understand. For some, there might even be a desire to return to the cult.

*‘And I felt bad about leaving, partly because I missed the feeling of belonging somewhere, the friends, the ones I liked, what was good, and I had a certain desire to return’ ID 10.*

They had difficulties explaining what they been through; they found they had no words to describe it. They perceived that others would not be able to deal with hearing about what had happened to them. They had a hard time being open about their backgrounds after leaving the cult, due to both shame about their history in the cult and emotional difficulty talking about their experiences.

*‘You cannot even find language for what you have been through; I think that I’ve never really talked to anyone about it because, the people I surrounded myself with afterwards did not understand this’ ID 4.*

*‘I did not tell anyone that I was a [name of cult] or that I had grown up there. It’s so hard to explain, so I just made it up that I had gone to boarding school’ ID 6.*

### Needs and meaning making

Former cult members described their needs after leaving the cult, and their ways of making new meaning in order to move on with their lives.

When they contacted the healthcare services, they experienced that religious and existential matters were not acknowledged. They stated that medical treatment with antidepressants and anxiolytics only partially helped. However, those who received trauma-focused psychotherapy found it helpful and life changing. Equally important was finding a community of other former cult members who had had similar experiences. Finding and meeting others like them and reading others’ written accounts of their experiences was felt to be helpful.

*‘It feels like one thing does not rule out the other, it’s just as important. I feel that the eye movement therapy*^1^ *saved my life. Thanks to the trauma therapy, I can live and feel that I have a normal quality of life. […] However, all the support I’ve received in the former cult member community, both on Facebook and YouTube, is at least as important’ ID 5.*

They felt a sense of belonging with others who shared their experiences. For those former cult members who had friends or family outside the cult, these relationships had the potential to give new meaning to their lives during periods when finding meaning was difficult. Trusting others was difficult, but this became easier when they felt genuine support from others.

*‘My friends and the relations that I thought I’d lost a long long time ago when I joined [name of cult] are coming back. It is like something is happening, life is coming back’ ID 11.*

They found a new sense of joy in the simple things in life. They described their new view of life in terms of having more faith in humanity after leaving the cult, and the belief that goodness and good people exist in the world. They now felt that they were being guided by common sense and no longer being ruled by someone else. They found that they were open to new opportunities. They now described cult life in terms of having been spiritually abused and constrained.

*‘When I was in the group, my view of the world was very dark. I was convinced that humanity would not be able to survive. That we were on the brink of doom. I only saw evil, I only saw horrors, focused only on the bad news. [...] But today I see positive things, today I believe in humanity. […] And I enjoy living. I did not then. It’s because now I look hopefully at the future. For myself and for people in general’ ID 3.*

### Persistent problems

Former cult members received various kinds of help and support. Despite the help they received, several of them still had persistent problems after some time of trying to manage their new life. They experienced grief at the loss of friends who were still in the cult, as well as depression, anxiety, fear of starvation and phobias.

*‘It’s hard to have this anxiety backpack, and carry all the sadness, all the memories. […] I do not think I would be where I am today without it. I think it [the cult] has absolutely affected me negatively. It’s like I’m free even though I’m not free and that’s so frustrating. It’s as if they still have a hold on me’ ID 8.*

Some of them also continued to self-harm and had alcohol problems. They sometimes experienced somatic symptoms and did not look after their bodies.

*‘[There have been] a lot of problems. I’ve had alcohol problems that I got help with a little bit more than a year ago. And that was my way to self-harm to handle my anxiety. […] I do not know, I am just very insecure, scared, and anxious’ ID 1.*

Other persistent problems included nightmares, high arousal, and feeling triggered by anything that reminded them of the cult. They knew they were free having left the cult, and yet they did not feel free. Just getting through the day could involve a lot of hard work.

*‘I have a lot of nightmares about people screaming at me, because they screamed a lot at you [in the cult] and you were scared all the time. I have nightmares about the end of the world, and that it’s my fault. And at the same time, I avoid feeling it and thinking about it’ ID 6.*

## Discussion

After leaving the cult, former cult members face a period they experience as an ‘in-between time’: no longer part of the cult, but not yet fully part of mainstream society. During this time, they have problems understanding their thoughts and feelings. Their former worldview is no longer valid when they enter the mainstream – in this case, Swedish secular society – and they lack a functioning worldview. They experience severe disruptions in existential meaning, similar in many ways to experiences described by individuals from other minority culture groups who have been through trauma (16). They feel they do not belong anywhere, and they are tasked with restoring and/or building a new worldview. This is a period of chaos and crisis, which is nonetheless often followed by a time when they feel more able to connect to themselves and others. They can then find a sense of belonging and new meaning. However, some former cult members do continue to suffer from mental distress. The cultural environment in the cult is highly controlling and abusive, but it also provides a sense of belonging and existential meaning. The members experience love within the cult and they lose friends and in some cases family members upon leaving or being excluded from the cult. This adds stressors for them when they leave the cult. As Gibson et al. (28) states, being raised in a cult can instill social confidence and a sense of belonging even when cult members have been abused. Due to difficulties resolving these contradictory feelings and the puzzle of identity, religion/ideology, and faith, it is hard for former cult members to understand and express their experiences. This make it harder for them to leave the cult mentally and find a new direction in life. Learned shame and isolation from self is an issue in cults (9, 10), which is reflected in this study. The former cult members state that they were not allowed to have their own feelings and were in a constant struggle to do everything right according to the cult values. Cult ideology can give meaning to and provide explanations for traumatic experiences in the individual’s life (in some cases, events that happened even before joining the cult), and to leave the cult and its ideology behind can disrupt this explanatory system, which has thus far served as a narrative of the person’s life. Therefore, measures should be taken in health care settings to strengthen the person’s inner dialogue to break the isolation from self, and screen for possible traumas that may have occurred both in the cult and before joining the cult (29). The cult environment is often described by former members as an honor culture and as condoning sexual abuse by cult leaders, which is in stark opposition to mainstream, secular Swedish society which insists on gender equality (18). What some of the respondents refer to as ‘acts of honor’ or ‘an honor culture in the cult’ refers to their experiences of feeling that there was a strict sense of control over them and in their contacts with people outside the cult, especially with persons of the opposite sex. In addition there is also mentioned, a demand from other cult members for them to engage themselves more deeply in the ideology was important for avoiding violating the honor of the cult. According to Boeri (30), being female may increase vulnerability, difficulties with acculturation, and exposure to traumatic events both in the cult and after leaving.

Former cult members manage to find new meaning by meeting others who share similar experiences; this can help them to make sense of what they have been through. It has also previously been stressed as crucial for former cult members to meet others who share similar experiences (31) and that support groups can provide relief from psychological difficulties after leaving a cult (32). Psychoeducation can also be important for helping former cult members understand their experiences and reactions after leaving a cult (31, 33). Other close relationships like those with friends and family are also important for recovery. Through these experiences, former cult members can start to feel trust again and to believe in themselves, find new ways of making meaning in life, and connect to the wider society outside the cult, thus ending their isolation and connecting to themselves and others. They also describe a new life post cult that is guided by their own values, common sense and finding meaning in the small things in life. They create a new narrative of their life and who they are.

For former cult members to decide what to retain from their old culture and what to adopt from their new culture is part of the acculturation process. This can be challenging as the culture of the cult will often have been highly demanding and often abusive, in contrast with the newly found freedom in the mainstream society. There were some former cult members in the study who had not been able to get help or find adaptive ways of managing the acculturation process or handling their traumas. They experienced nightmares, high arousal and avoidance, which could be symptoms of PTSD, as well as anxiety, depression, cult-related phobias (9), prolonged grief and somatic complaints. Thus, it is important to evaluate the risk factors for developing PTSD (34) and other mental disorders. An awareness of potential sexual abuse that may have occurred and been mistaken for consensual sex must also be acknowledged due to the loss of autonomy that occurs in the cult (35). The many persistent symptoms of mental distress found in former cult members also indicate the frequency of traumatic events and the loss of power that individuals experience both while they are members and after leaving the cult. The trauma bond and disorganization of self often seen in former cult members could be largely due to the early, multiple and interpersonal trauma experiences that so often characterize life in the cult.

In acculturation research, it has been shown that it is important how the new culture’s healthcare representatives interact with patients. This includes conveying respect and care, and can play a key role in decreasing the patient’s trauma symptoms and increasing their level of trust in the healthcare system (36, 37). Healthcare professionals thus play an important role in representing the majority culture and the society that former cult members encounter outside the cult.

Healthcare providers must offer help in the form of adaptive coping strategies to facilitate acculturation, and adopt a person-centered and culturally aware approach. Studies have shown better outcomes and compliance when such strategies are used (38). Healthcare professionals with no formal training in cultural competence, including existential matters, risk being unable to use this approach (2, 39). Health care has the potential both to improve patient health and to inadvertently make it worse by misunderstanding the cultural context because of a lack of training.

All patients are individuals, and all patients have their particular culture. Cultural awareness among healthcare practitioners may be particularly important when meeting former cult members, since cult experiences appear to have such a deep and lasting impact on sense of self, trust and relationships, and given the highly likely presence of trauma. This became clear in this study and is consistent with previous findings in this field (8, 40, 41).

From the perspective of the sections of the CFI, the results show *‘cultural definition of problem’* and *‘cultural perceptions of cause, context and support’* as being closely linked to a starting point in the worldview derived from the cult that eventually changed in the new cultural setting into which former cult members were acculturated, i.e., the majority culture of mainstream, secular Swedish society. It was also revealed how some former cult members created a new minority culture together with other former cult members, defining their problems and perceptions of cause in this setting. The questions in the CFI regarding *‘cultural factors affecting self-coping and past help seeking’* and *‘cultural factors for current help seeking’* revealed a variety of variables affecting help seeking among former cult members. As stated in earlier research into former cult members’ perceptions of healthcare consultations (42), help seeking appears to be affected by how the cult ideology views seeking help, what learned reasons the respondents had for not feeling well, and a reduced ability to connect with their own values and feelings. Overall, the ability to seek help appeared to increase for most respondents when they managed to overcome the in-between time and become more aware of their needs.

### Strengths and limitations of this study

The use of the CFI as an interview guide was well accepted and the former cult members described many existential aspects of their lives and their acculturation process during and after leaving their cult. They described both meaningful and helpful factors on their journey, as well as problems as perceived from their own perspective. Their accounts were rich, open-hearted and full of trust. It appeared as if they felt they had been listened to. Using the CFI allowed for accounts to be retrieved in a semi-structured way with a great deal of respect and sensitivity being shown to former cult members. It also provided deep insights into former cult members’ perceptions of their problems, needs, meaning making and the acculturation process.

By using systematic text condensation, starting by gaining an overall impression of the entire material and forming preliminary themes, it was possible to discern how the process of acculturation was described by the respondents at an early stage of the analysis. Thus, it was possible to identify the lived experience of their narratives about their transformation – not just moving from one culture to another, but being in between and in several cultures simultaneously. This combination of using a well-researched interview format with questions facilitating a culturally aware approach such as the CFI, together with an analytical method that facilitated an overview of and closeness to the data, produced valuable new knowledge. Another possible method could have been to analyze the responses to the CFI on an area-by-area basis. However, the decision was made not to adopt this approach but rather to stay closer to the way the respondents expressed their narratives chronologically. In this way, the CFI served as a tool for interviewing and collecting data but not for steering the process of analysis in any particular direction.

Credibility and trustworthiness in this study stem from a rigorous analysis close to the respondents’ own words with help from quotes and the method of condensation that serves to ensure that all expressed experiences in a subcategory are coherent. Another strength is the intense co-operation in the research group behind the study, which comprises specialists in different clinical and research fields with broad competences. This study provides an in-depth examination of a vulnerable population that is of relevance for both cultural- and transcultural psychiatry as well as for mental health contexts in general in Sweden. Even though this is a Swedish study, it may be useful and applicable to other populations and patient groups seeking healthcare in other societies.

The former cult members in this study are an example of individuals with an invisible cultural context. Cult members/former cult members as well as potentially other cultural minority groups may not have visible attributes, e.g., a different language or appearance, since other features could define their culture. The study points to the importance of understanding that acculturation processes may occur among individuals with no visible or obvious attributes that distinguish them from others in the mainstream, secular society. This challenges healthcare providers to identify the landscape ‘beneath the snow’, since this is where understanding of their patients can be gained.

The study included more female than male respondents, which could have resulted in more reports of specific kinds of abuse (i.e., psychological, sexual). However, no obvious differences were observed regarding the sex of the respondent. The former cult members in this study were recruited *via* social media; it is possible that respondents who are active on social media and in Facebook groups are in greater need of post-cult support.

A broad variation of time since leaving the cult exists among the participants of this study. To some extent a variation in their stories was identified according to how long it was since they left their cult. A progression in the acculturation process is visible in the individual stories of the respondents as well as in the dataset as a whole. However, no specific patterns of time laps or events were detectable in this study. The broad range of experiences and of time laps since leaving the cults were considered helpful and enriching for the depth of the analysis of the data, and facilitated an overview of the processes involved when leaving a cult situation.

## Conclusion

Former cult members face a challenging acculturation process after leaving the cult, having lost a functioning worldview but not yet gained another to take its place. While this ‘in-between time’ is often transient, they may need support from the healthcare system, especially regarding mental health concerns, while establishing themselves into mainstream society.

## Data availability statement

The original contributions presented in the study are included in the article/supplementary material, further inquiries can be directed to the corresponding author.

## Ethics statement

The studies involving humans were approved by Swedish Ethical Review Authority; Regional Ethic board in Umeå. The studies were conducted in accordance with the local legislation and institutional requirements. The participants provided their written informed consent to participate in this study.

## Author contributions

CH, OS, and AL did the code process discussed with all the authors. CH wrote the manuscript. All authors have participated in designing the study and contributed equally to the process of writing and discussing the content.

## Conflict of interest

The authors declare that the research was conducted in the absence of any commercial or financial relationships that could be construed as a potential conflict of interest.

## Publisher’s note

All claims expressed in this article are solely those of the authors and do not necessarily represent those of their affiliated organizations, or those of the publisher, the editors and the reviewers. Any product that may be evaluated in this article, or claim that may be made by its manufacturer, is not guaranteed or endorsed by the publisher.

## Footnotes

^1^“Eye Movement Desensitization and Reprocessing Therapy” (EMDR).
